# Combined lactate- and phosphate-dependent cytoplasmic acidification is associated with *Mycobacterium tuberculosis* growth arrest at acidic pH

**DOI:** 10.1371/journal.pgen.1012331

**Published:** 2026-10-09

**Authors:** Adam P. Kibiloski, Shelby J. Dechow, Bassel J. Abdalla, Heather M. Murdoch, Anna D. Tischler, Robert B. Abramovitch

**Affiliations:** 1 Department of Microbiology, Genetics, and Immunology, Michigan State University, East Lansing, Michigan, United States of America; 2 Department of Microbiology and Immunology, University of Minnesota, Minneapolis, United States of America; Texas A&M University, UNITED STATES OF AMERICA

## Abstract

*Mycobacterium tuberculosis* (Mtb) cultured in minimal medium at acidic pH arrests growth when provided specific single carbon sources, including glycerol, propionate, and lactate, a phenomenon we refer to as acid growth arrest. To define the mechanisms of acid growth arrest on lactate, we selected transposon mutants that grew under these conditions. Five mutants carried insertions in *phoT or pstC2*, which encode components of a phosphate ABC transporter. Depleting phosphate restored Mtb growth in minimal medium supplemented with lactate at acidic pH, demonstrating that growth arrest on lactate depended on phosphate. The combination of lactate and phosphate at acidic pH acidified the cytoplasm to below pH 6.7, whereas the *phoT* mutant maintained a cytoplasmic pH > 7.2. Lactate slightly increased membrane potential in wild-type Mtb, whereas the *phoT* mutant maintained a higher membrane potential. Transcriptional profiling identified a lactate-associated PMF stress signature, including upregulation of electron transport chain genes. Together, these findings associate lactate- and phosphate-dependent cytoplasmic acidification with proton motive force (PMF) stress and acid growth arrest. The *phoT* mutant grown in lactate at acidic pH upregulated the *senX3*/*regX3* regulon. Using a *regX3* mutant, we found that growth on lactate under low-phosphate conditions required *regX3*. We propose a model in which (1) acidic pH, lactate, and phosphate together modulate cytoplasmic pH homeostasis and are associated with PMF stress and acid growth arrest; and (2) low phosphate induces SenX3-RegX3 activity, which may alter cell-envelope physiology through ESX-5- and PE/PPE-dependent pathways and promote growth on lactate at acidic pH.

## Author summary

*Mycobacterium tuberculosis* (Mtb) grows well on lactate as a sole carbon source at neutral pH but not at acidic pH. This study investigated the basis of this pH-dependent growth restriction. A genetic selection for mutants that grew on lactate at acidic pH identified insertions in genes encoding phosphate-transporter components. We found that depleting extracellular phosphate or disrupting a phosphate-transporter component promoted growth on lactate at acidic pH and that this growth required the phosphate-responsive two-component regulatory system SenX3-RegX3. We further found that lactate, phosphate, and acidic pH acted together to acidify the cytoplasm, a metabolic stress associated with acid growth arrest on lactate.

## Introduction

*Mycobacterium tuberculosis* (Mtb) is an intracellular bacterial pathogen that survives and replicates in macrophages. After phagocytosis, Mtb encounters mildly acidic pH in the macrophage phagosome [1]. Mtb must sense and adapt its physiology to acidic pH to remain virulent in macrophages and animal models [2–7]. In defined minimal medium containing a single carbon source at acidic pH, Mtb either grows or establishes a state of nonreplicating persistence, depending on the carbon source [8–11]. For example, Mtb grows relatively well in minimal medium buffered at pH 5.7 and supplemented with permissive carbon sources that feed the anaplerotic node, such as pyruvate or oxaloacetate [8]. In contrast, Mtb enters a nonreplicating state that we refer to as acid growth arrest when cultured at pH 5.7 with nonpermissive carbon sources such as glycerol, lactate, or propionate [10].

The acid growth arrest phenotype was initially perplexing because Mtb has everything needed to grow under these conditions, including oxygen as a terminal electron acceptor and a carbon source that supports growth at pH 7.0. We therefore hypothesized that acid growth arrest is regulated and that genetic mutations could suppress it. To test this hypothesis, we selected mutants that grew on glycerol as the sole carbon source at acidic pH. We termed this phenotype Enhanced Acid Growth (EAG), and the selection demonstrated that genetic mutations could overcome growth restriction on glycerol [12]. All isolated EAG mutants carried dominant mutations in *ppe51* that increased glycerol uptake and enhanced replication both *in vitro* and in activated macrophages [12]. Notably, enhanced replication in macrophages reduced fitness, supporting the conclusion that Mtb must integrate acidic pH and carbon source utilization to regulate growth and promote pathogenesis in macrophages. We also isolated suppressors of acid growth arrest on propionate as the sole carbon source; all carried mutations in the *phoPR* two-component system [13]. Genetic and biochemical studies showed that diversion of propionate toward methyl-branched lipid synthesis caused growth arrest on propionate. Thus, distinct, carbon-source-specific stresses underlie acid growth arrest on glycerol and propionate. Moreover, *ppe51* EAG variants and *phoPR* mutants did not grow on lactate at acidic pH [12,13], suggesting that a third mechanism underlies acid growth arrest on lactate.

Mtb catabolizes lactate to promote growth in human macrophages [14], demonstrating the importance of lactate during infection. Other pathogens, including *Neisseria spp*., *Haemophilus influenzae*, and *Staphylococcus aureus*, also use host-derived lactate to promote pathogenesis [15]. In this study, we sought to define the mechanism of acid growth arrest in Mtb cultured at acidic pH with lactate as the sole carbon source. We selected transposon mutants that grew on lactate at acidic pH and identified four mutants with insertions in *phoT* and one with an insertion in *pstC2*. PhoT and PstC2 are components of the PstSCA-PhoT phosphate ABC transporter, supporting a link between phosphate and growth arrest on lactate. We found that growth arrest on lactate depended on phosphate concentration and the SenX3-RegX3 two-component regulatory system. We also found that lactate and phosphate were associated with cytoplasmic acidification and PMF stress. Together, the findings of Tischler *et al*. [16], Healy *et al*. [17], and this study reinforce the critical role of phosphate in regulating pH-dependent adaptations.

## Results

### Mtb remains viable but does not replicate on lactate at acidic pH

Previous studies showed that wild-type (WT) Mtb does not grow on lactate at pH 5.7 [8,12]. In defined medium containing lactate as the sole carbon source, WT Erdman grew at pH 7.0 but arrested growth at pH 5.7 (Fig 1A). We hypothesized that the arrested bacteria remained viable at pH 5.7 but did not replicate. After nine days of growth arrest on 4 mM lactate, we added the permissive carbon source pyruvate. The bacteria resumed replication, demonstrating that they remained viable on lactate at pH 5.7 (Fig 1A). We confirmed viability by enumerating Mtb colony-forming units (CFUs) and observed no reduction in CFUs over time (Fig 1B). WT CDC1551 showed the same growth pattern (S1A Fig). Therefore, acid growth arrest on lactate represents a state of nonreplicating persistence.

**Fig 1:**
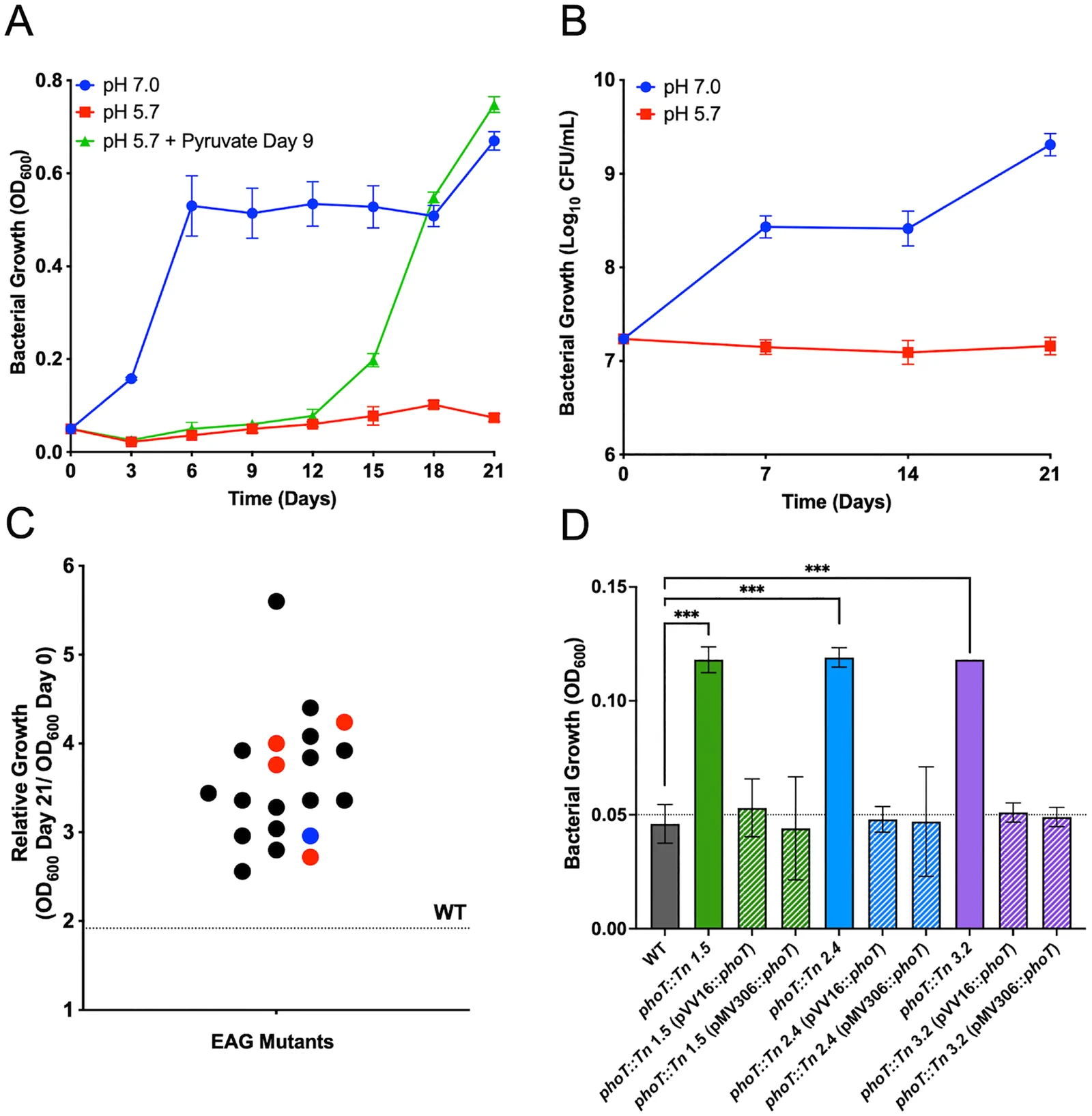
Selection of *phoT* mutants that suppress acid growth arrest on lactate. A. WT Erdman was grown in minimal medium buffered to pH 5.7 (red) and 7.0 (blue) supplemented with 4 mM lactate. On day 9, 10 mM pyruvate was added to Mtb grown on lactate at pH 5.7 (green). This experiment was performed at least twice with similar results. Error bars indicate standard deviation of three technical replicates. B. WT Erdman was grown in minimal medium buffered to pH 5.7 (red) and pH 7.0 (blue), supplemented with 4 mM lactate. This experiment was performed at least twice with similar results. Error bars indicate the standard deviation of three technical replicates. C. Confirmation of suppressor mutants by examining relative growth at Day 21 OD_600_ to OD_600_ from the initial Day 0 inoculum. Each dot represents a selected transposon mutant isolated from MMAT agar plates and confirmed for enhanced growth in liquid MMAT (pH 5.7) supplemented with 4 mM lactate. Red dots indicate *phoT::Tn* mutants, the blue dot represents a *pstC2::Tn* mutant. The dotted line indicates the relative WT Erdman growth (> ratio of 1). This experiment was performed once with a single replicate. D. Three of the *phoT::Tn* mutants were complemented using a single copy chromosomal insertion vector (pMV306) or an episomal overexpression vector (pVV16). Black bar indicates growth arrest in WT. Data is OD_600_ after 21 days of growth. Statistical analysis was performed using an unpaired t-test (***, p < 0.001). This experiment was performed once. Error bars indicate the standard deviation of three technical replicates.

### A transposon selection identifies mutants that suppress growth arrest on lactate at pH 5.7

In minimal medium at acidic pH with lactate, Mtb has both a carbon source and oxygen and therefore appears to have everything needed for growth. We hypothesized that acid growth arrest on lactate is a regulated adaptation and that genetic mutations could suppress it. We plated an Mtb Erdman transposon library containing >100,000 mutants at a density of 10^9^ CFUs per plate. After eight weeks, > 1,000 colonies grew across four plates, and we randomly selected 20 for confirmation. All 20 mutants grew more than WT Erdman on lactate at pH 5.7; WT Erdman remained growth arrested (Fig 1C and S1B Fig). We identified the transposon insertion sites in 15 mutants by inverse PCR and Sanger sequencing or by whole-genome sequencing [18]. Four independent mutants carried transposon insertions in *phoT* (Rv0820), which encodes the ATP-binding protein of a phosphate ABC transporter, and one carried an insertion in *pstC2* (Rv0929), which encodes an integral membrane component of a phosphate ABC transporter (Table 1). We complemented the *phoT::Tn* strains by expressing WT *phoT* from either a single-copy chromosomal insertion vector (pMV306::*phoT*) or an episomal overexpression vector (pVV16::*phoT*). In minimal medium at acidic pH supplemented with 4 mM lactate, *phoT::Tn* mutant strains 1.5, 2.4, and 3.2 grew, whereas WT and all complemented strains remained growth arrested (Fig 1D). Thus, loss-of-function transposon insertions in *phoT* promoted growth on lactate. We used *phoT::Tn* strain 1.5 for the remaining studies unless otherwise specified.

**Table 1:** Transposon insertion sites that permit growth on lactate. Mutants were arbitrarily assigned identification numbers based on the order they were picked from the plate. FC indicates the fold-change increase in growth relative to WT on lactate. TA site indicates the TA in which the transposon recognized and inserted. The numbers indicate the nucleotide number the TA is at over the total nucleotide length of the gene. The method of identification is either whole-genome sequencing (WGS) or inverse PCR as indicated.

Mutant #	Insertion Start Site	Insertion Stop Site	Approx TA site/gene length (nt)	FC relative to WT growth	Rv Number	Gene Name	Method of identification
1.5	916505	916506	TA 197/777	2.72	Rv0820	*phoT*	Inverse PCR
2.4	917077	917078	TA 770/777	3.76	Rv0820	*phoT*	WGS
3.2	917060	917061	TA 754/777	4.24	Rv0820	*phoT*	Inverse PCR/WGS
4.2	917010	917013	TA 703/777	4.00	Rv0820	*phoT*	WGS
4.5	1040405	1040406	TA 690/975	2.96	Rv0929	*pstC2*	WGS

### The *phoT::Tn* mutant grows on lactate at acidic pH

We previously found that *ppe51* variants and *phoPR* mutants showed selectively enhanced growth on glycerol and propionate, respectively, but did not grow on other nonpermissive carbon sources, including lactate [12,13]. We hypothesized that the *phoT::Tn* mutant would also show carbon-source-specific enhanced growth at acidic pH. We therefore grew WT, *phoT::Tn*, and complemented strains in minimal medium at acidic pH with individual carbon sources. The *phoT::Tn* mutant grew only on lactate at acidic pH and reached ~8-fold greater growth than WT (Fig 2A). These findings support the conclusion that lactate-specific physiology contributes to growth arrest at acidic pH.

**Fig 2:**
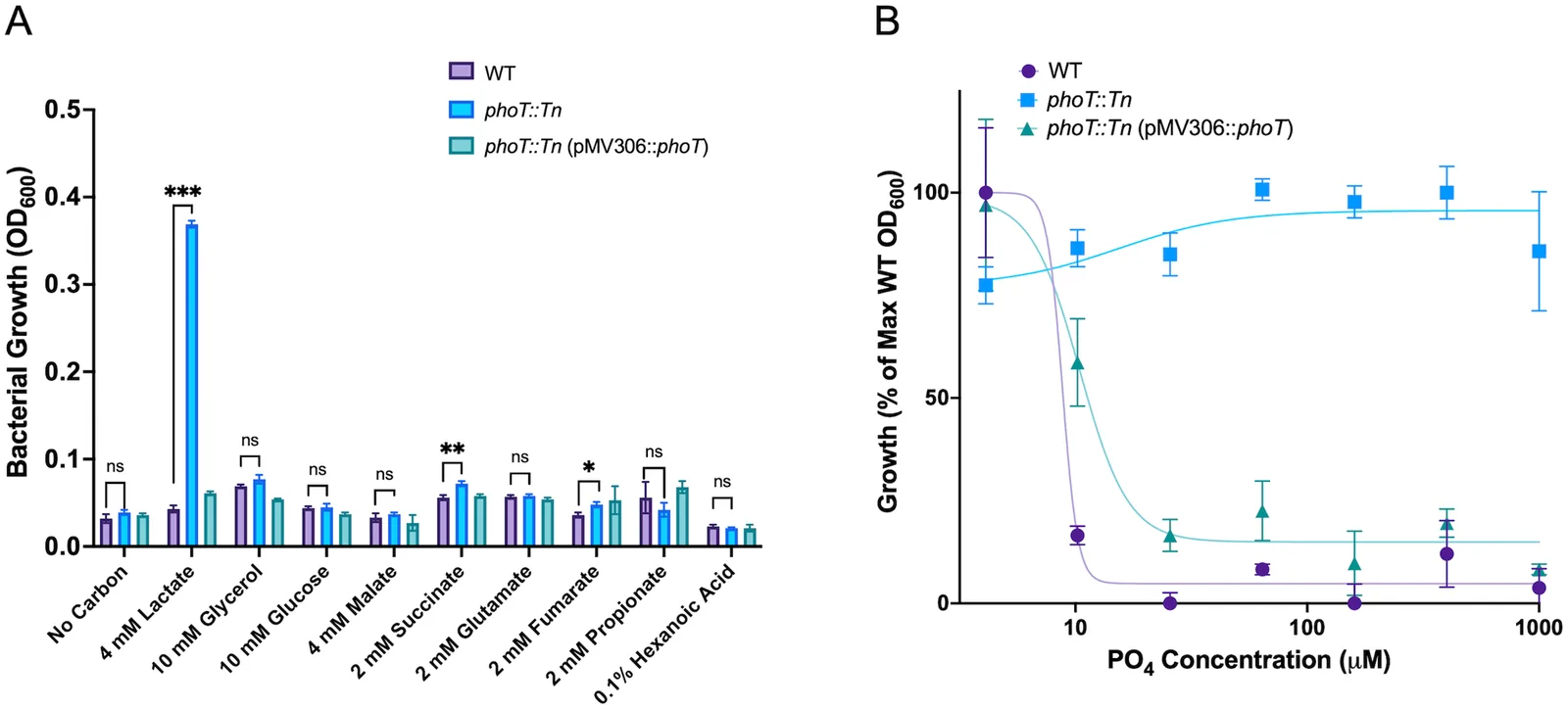
The *phoT*::Tn mutant grows selectively on lactate across phosphate concentrations. A. Growth of the WT, *phoT::Tn* mutant and the complemented strains cultured in a variety of host associated carbon sources. Data measured on day 21 with no growth indicated by the “No Carbon” control. Statistical analysis was performed using an unpaired t-test (*, p<0.05, **, p < 0.005, ***,p < 0.001). This experiment was performed at least twice with similar results. Error bars indicate the standard deviation of three technical replicates. B. WT (purple), *phoT::Tn* (blue), and *phoT::Tn* (pMV306*::phoT*) (teal) strains were grown in MMAT with lactate as the sole carbon source at pH 5.7 across a 2.5-fold dose response of KH_2_PO_4_. Values were normalized to percentage of maximum WT growth. This experiment was performed twice with similar results. Error bars indicate the standard deviation of three technical replicates.

### Phosphate limitation restores Mtb growth on lactate at pH 5.7

We hypothesized that the *phoT*::*Tn* mutant may have altered phosphate uptake or phosphate-responsive signaling and that low phosphate may promote growth on lactate at acidic pH. We therefore grew WT, *phoT::Tn*, and complemented strains at pH 5.7 with 4 mM lactate and a serial dilution of monopotassium phosphate (KH_2_PO_4_) from 4.1 µM to 1.0 mM, including a no-phosphate control. Standard MMAT minimal medium contains ~25 mM phosphate. The *phoT::Tn* mutant grew well at all tested phosphate concentrations (Fig 2B). WT and complemented strains arrested growth at phosphate concentrations at or above 10 µM but grew as well as the *phoT::Tn* strain at 4.1 µM phosphate and in the no-phosphate control (Fig 2B). Low phosphate similarly promoted growth of H37Rv on lactate at acidic pH (S2 Fig). To determine whether potassium affected these growth phenotypes, we compared sodium phosphate with potassium phosphate. We grew WT Mtb, *phoT::Tn*, and the complemented strain in complete MMAT (containing sodium and potassium phosphate), MMAT without phosphate, MMAT containing only potassium phosphate, or MMAT containing only sodium phosphate. After 21 days, WT remained growth arrested whenever phosphate was present, regardless of the counterion (S3 Fig). The *phoT::Tn* strain grew ~1.6-fold less in MMAT containing sodium phosphate than in MMAT containing potassium phosphate (S3 Fig), indicating a counterion-dependent difference in growth whose mechanism remains unclear. Overall, these data show that phosphate restricts growth on lactate at acidic pH, whereas disrupting *phoT* or depleting phosphate from the medium overcomes this restriction.

We hypothesized that phosphate and lactate function together to regulate growth at acidic pH and sought to define the concentrations that permitted or restricted growth. We performed checkerboard assays across 2.5-fold serial dilutions of lactate (164 µM to 40 mM) and phosphate (0.8 µM to 200 µM), including a no-phosphate control. Strains grew only at lactate concentrations above 1 mM and up to 6.4 mM (Fig 3), indicating a narrow permissive range. Lactate concentrations >6.4 mM likely inhibited growth because of toxicity [19], whereas concentrations at or below 1.0 mM did not support growth. At >12 µM phosphate, only the *phoT* mutant grew on lactate (Fig 3). At <12 µM phosphate, however, WT Mtb grew at 6.4 mM lactate. Thus, phosphate and lactate concentrations together determined acid growth arrest. We also assessed pH dependence by growing WT, *phoT::Tn*, and pMV306-complemented strains with 4 mM lactate across pH 5.5-7.0. The *phoT::Tn* mutant grew at pH 5.5-6.0 and grew most robustly at pH 6.0, where it reached 10-fold greater growth than WT (S4A Fig). All strains grew robustly at pH 6.5 or greater. Together, these data show that lactate concentration, phosphate concentration, and acidity interact to regulate growth.

**Fig 3:**
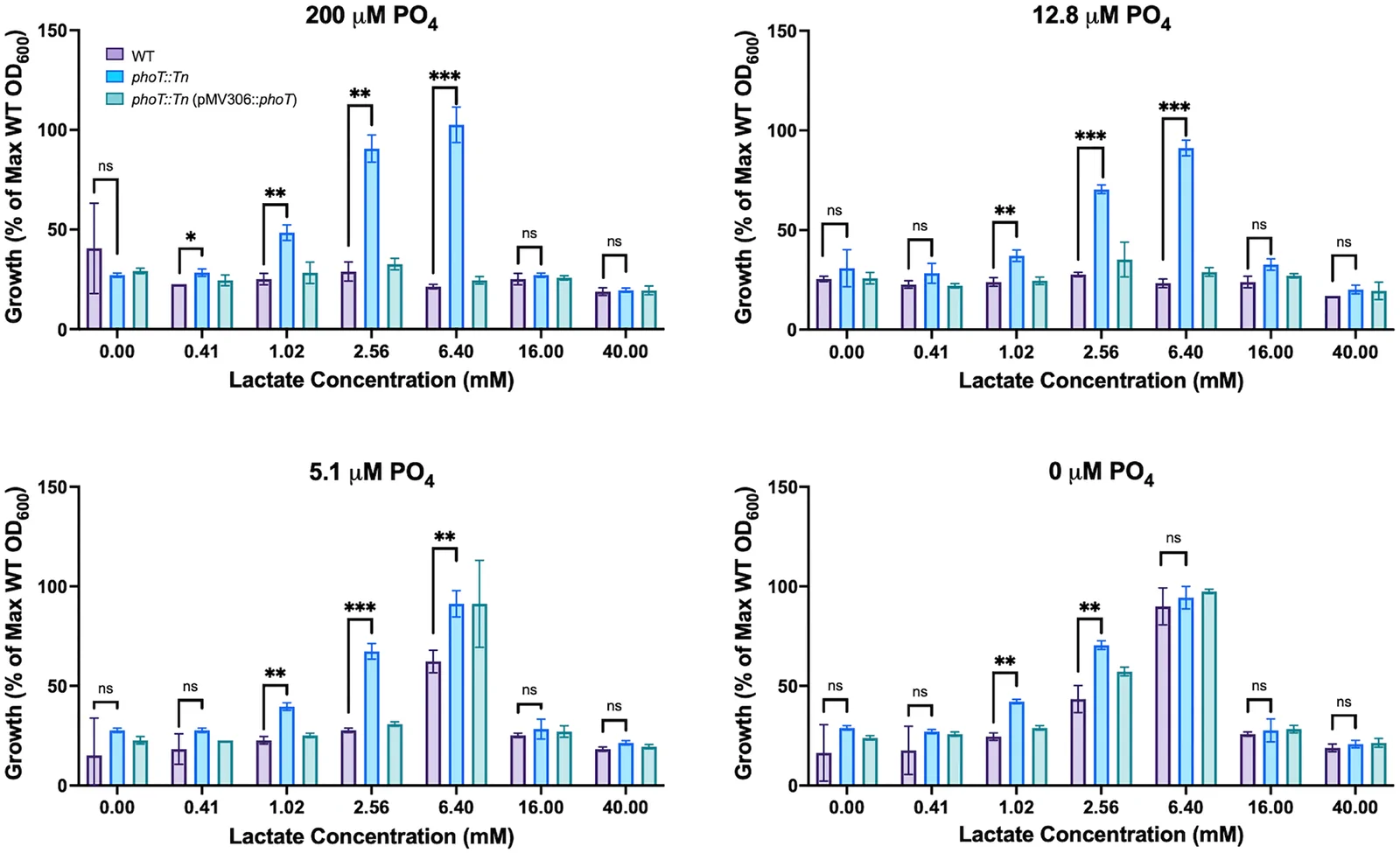
Mtb growth arrest on lactate is dependent on phosphate concentration. Mtb grown at varying lactate concentrations in media amended with (A) 200 µM, (B) 12.8 µM, (C) 5.1 µM, and (D) with no PO_4_. Data is reported as % of maximum of WT growth (observed in the 0 µM phosphate condition). Statistical analyses were performed using an unpaired t-test (*, p < 0.05, **, p < 0.005 ***, p < 0.001) This experiment was performed twice with similar results. Error bars represent the standard deviation of three replicates.

### Lactate and phosphate act together to acidify the cytoplasm

Even under acidic conditions, Mtb maintains a relatively neutral cytoplasmic pH of ~7.2-7.4 [5,8]. Loss of cytoplasmic pH homeostasis is associated with cell death, possibly through disruption of PMF or protein unfolding [5,20]. High lactate concentrations expose lactic acid bacteria to several stresses, including cytoplasmic acidification and altered oxidative phosphorylation due to PMF stress [21,22]. Because protonated lactic acid can release a proton after entering the cytoplasm and phosphate uptake can affect pH homeostasis or its energetic demands, these nutrients could alter cytoplasmic pH or membrane potential. We therefore hypothesized that lactate or phosphate exposure acidified the Mtb cytoplasm or increased membrane potential. We measured cytoplasmic pH with the pH-sensitive fluorescent dye CMFDA [23]. We generated a standard curve by exposing Mtb to media buffered at different pH values and treating the bacteria with nigericin to equilibrate external and internal pH. We then measured cytoplasmic pH in Mtb incubated at pH 5.7 in minimal medium containing 0, 1, 4, or 10 mM lactate and either 0 or 200 µM phosphate. These conditions spanned growth-permissive and growth-arresting conditions identified in Fig 3. In the absence of phosphate, lactate acidified the cytoplasm in a dose-dependent manner (Fig 4). Cytoplasmic pH ranged from ~7.2 to ~6.8 when bacteria were incubated with 0, 1, or 4 mM lactate without added phosphate (Fig 4A- 4C); these conditions permitted growth (Fig 2B). At 10 mM lactate without phosphate, cytoplasmic pH decreased further to ~6.4 (Fig 4D), which may explain the growth restriction at high lactate concentrations (Fig 3). When we added 200 µM phosphate, Mtb maintained a relatively neutral cytoplasmic pH at 0 and 1 mM lactate (Fig 4A- 4B). In contrast, 4 mM lactate and 200 µM phosphate acidified the cytoplasm to below pH 6.5 (Figs 4C, S4C), conditions associated with growth arrest (Fig 2B). These pH values remained above the previously reported lethal cytoplasmic pH of <6.0 in *Mycobacterium smegmatis* and *Mycobacterium bovis* BCG [24]. Cytoplasmic acidification depended on lactate, where Mtb treated with glycerol or pyruvate maintained a cytoplasmic pH of ~7.4, consistent with our previous findings [8] and the repeated experiment shown here (S4B Fig). Thus, greater cytoplasmic acidification was associated with growth-restrictive conditions and was most pronounced when phosphate and lactate were combined.

**Fig 4:**
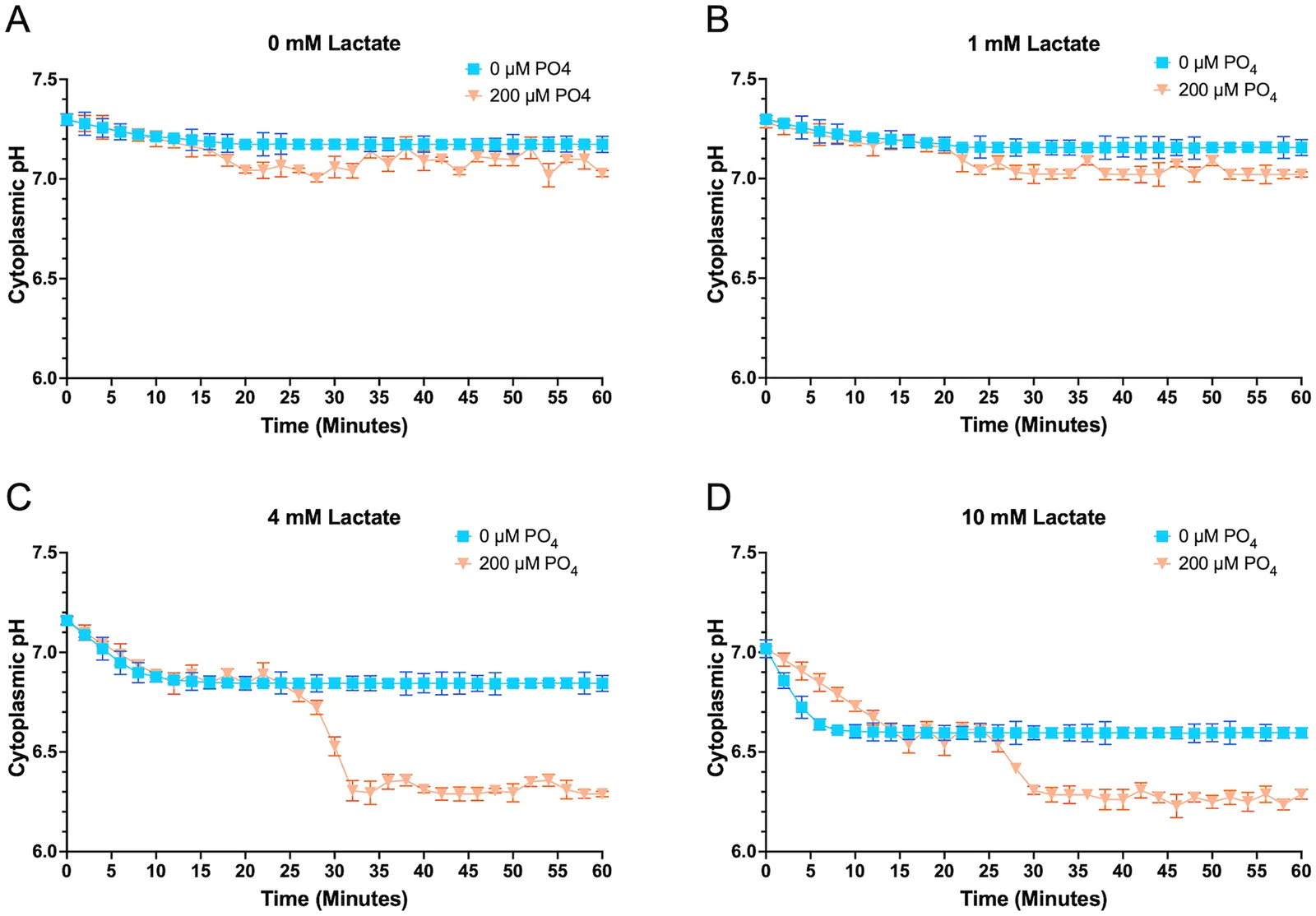
Lactate and phosphate together promote cytoplasmic acidification. Cytoplasmic pH was measured over time when exposed to 200 µM phosphate (orange), or no phosphate (light blue) in medium at pH 5.7 with no lactate (A), 1 mM lactate (B), 4 mM lactate (C), and 10 mM lactate (D). This experiment was performed at least twice with similar results. Error bars represent the standard deviation of three technical replicates.

We further hypothesized that altered phosphate uptake or signaling allowed the *phoT*::*Tn* mutant to maintain a more neutral cytoplasm. Using the same lactate and phosphate concentrations as in the preceding experiment, we measured cytoplasmic pH in WT, *phoT::Tn*, and *phoT::Tn* (pMV306:*:phoT*) strains. WT showed the same trend toward greater acidification at higher phosphate and lactate concentrations (Fig 5A). In contrast, the *phoT::Tn* cytoplasm was substantially less acidified (Fig 5B). The complemented strain reproduced the WT trend, except at 4 mM lactate without added phosphate, where it acidified (Fig 5C). Endpoint measurements at pH 5.7 showed that the *phoT* transposon insertion significantly reduced cytoplasmic acidification (Fig 5D). At extracellular pH 7.0, cytoplasmic pH remained above 7.2 in all strains (Figs 5E and S5). These data show that acidic pH, phosphate, and lactate were associated with greater cytoplasmic acidification and that mutating *phoT* alleviated this stress and suppressed acid growth arrest on lactate.

**Fig 5:**
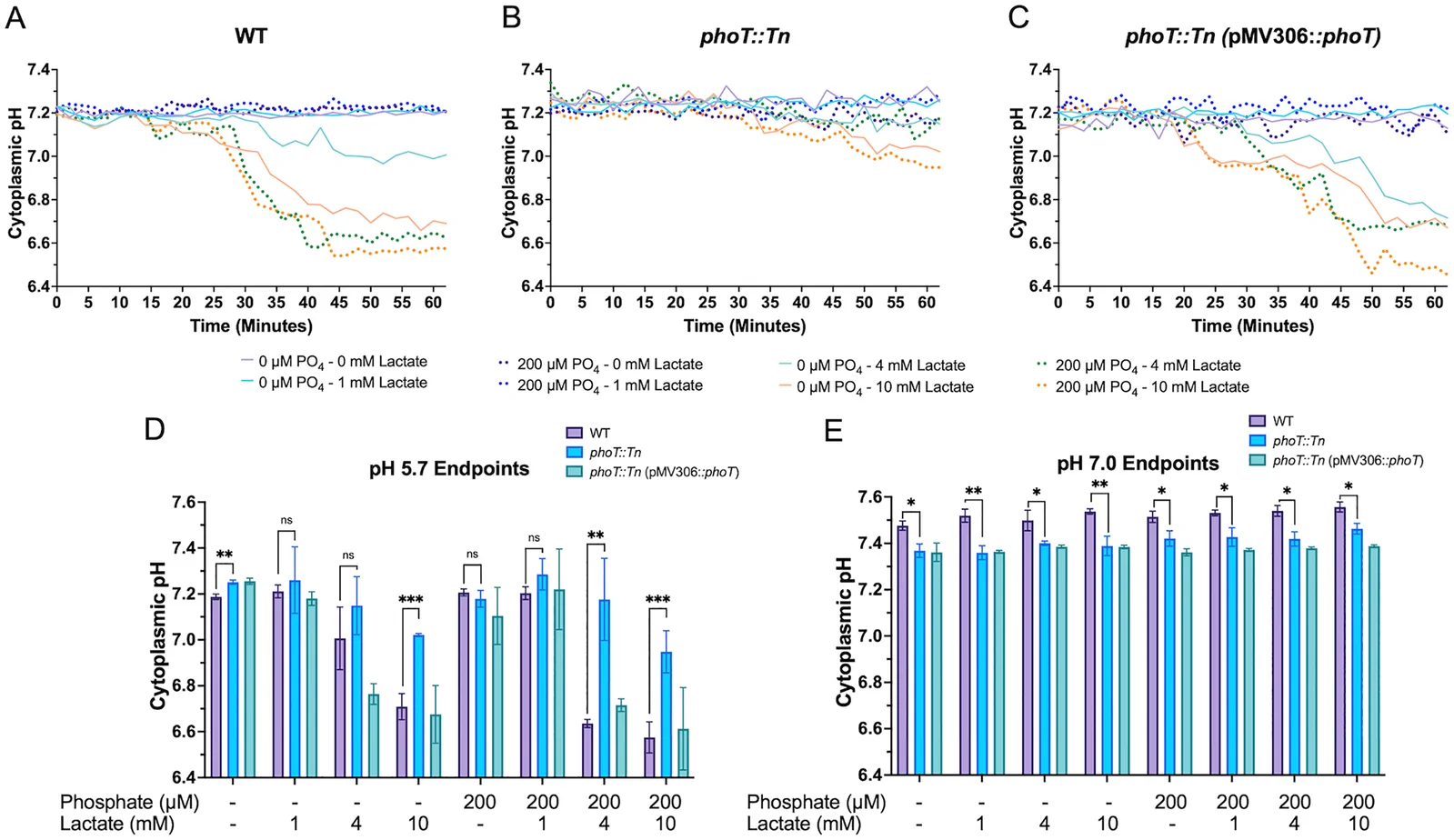
Disruption of *phoT* reduces cytoplasmic acidification. Cytoplasmic pH was measured over time in medium with the indicated range of phosphate and lactate concentrations for WT (A), *phoT*::*Tn* (B) and *phoT*::*Tn* (pMV306::*phoT*) (C) strains. Endpoint cytoplasmic pH (60 min) of strains exposed to lactate at pH 5.7 (D) or pH 7.0 (E). Statistical significance determined by an unpaired t-test (*, p<0.05, **, p < 0.005, ***, p < 0.001) All experiments for this figure were performed at least twice with similar results. Error bars represent standard deviation of three technical replicates.

### Lactate and disruption of *phoT* alter membrane potential

We observed significant cytoplasmic acidification when bacteria grew with 200 µM phosphate and 4 mM lactate but not with 1 mM lactate. This result suggested that Mtb maintained pH homeostasis at 1 mM lactate, possibly by exporting protons, which we predicted would increase membrane potential. We also predicted that disrupting PhoT-dependent phosphate transport or signaling would increase membrane potential. To test these hypotheses, we used the membrane-permeable fluorescent dye DiOC_2_ [25]. We measured membrane potential over time at pH 5.7 using lactate and phosphate concentrations similar to those in the cytoplasmic pH assay. Lactate at 1 and 4 mM modestly increased membrane potential, consistent with the hypothesis that Mtb exported protons to maintain cytoplasmic pH (Fig 6A-6B, S6A-S6B Fig). Unexpectedly, phosphate concentration did not strongly impact membrane potential at acidic or neutral pH (Fig 6A-6B and S6A-S6B Fig), suggesting that, under these conditions, phosphate affected the ΔpH component of PMF more strongly than membrane potential. At lower phosphate concentrations (0–200 µM), the *phoT::Tn* mutant showed only minor changes in membrane potential at acidic and neutral pH (S6A-S6B Fig). Thus, the *phoT::Tn* mutation did not substantially affect membrane potential at the tested lactate and phosphate concentrations in MMAT.

**Fig 6:**
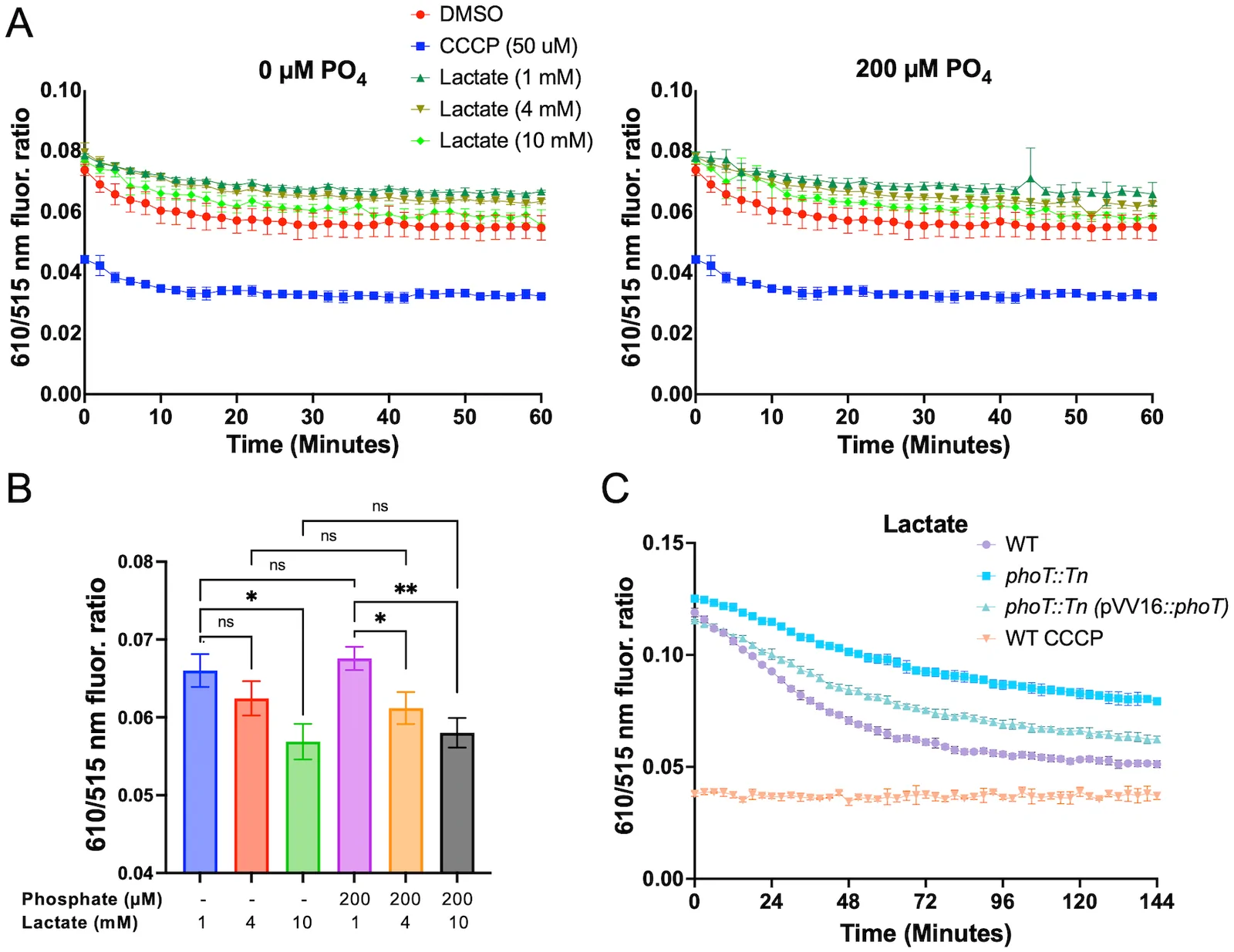
Lactate increases membrane potential and the *phoT::Tn* has higher membrane potential. A. Membrane potential was evaluated over time at 0 µM phosphate, and 200 µM phosphate. Lactate concentrations tested were 10 mM (light green), 4 mM (yellow-green), and 1 mM (forest green). A DMSO vehicle control was included (red). This experiment was performed twice with similar results. Error bars indicate the standard deviation of three technical replicates. B. Minutes 46–60 were averaged from panel A. These data represent endpoint data from the same experiment. C. Membrane potential of strains incubated in MMAT medium with lactate. The strains evaluated were *phoT::Tn* (pVV16::*phoT*) (teal), *phoT::Tn* (light blue), and WT (purple). WT treated with CCCP (orange) was used as a depolarized control. This experiment was performed at least twice with similar results. The error bars represent the standard deviation of three technical replicates.

To determine whether the potassium counterion affected membrane potential, we compared potassium phosphate with sodium phosphate. We measured membrane potential in WT, *phoT::Tn*, and the complemented strain over 60 minutes as the strains adapted to complete MMAT (containing sodium and potassium phosphate), MMAT without phosphate, MMAT containing only potassium phosphate, or MMAT containing only sodium phosphate. WT and the complemented strain had lower membrane potential with potassium phosphate than with sodium phosphate or complete MMAT (S7 Fig), suggesting that potassium phosphate lowered membrane potential at acidic pH. In the *phoT::Tn* mutant, membrane potential remained elevated with sodium phosphate and increased in potassium-phosphate-only medium to the level observed in complete MMAT. Thus, membrane potential depended on the phosphate counterion and *phoT* genotype under these conditions, but these data do not establish an underlying mechanism.

We also measured membrane potential in WT, *phoT*::*Tn*, and complemented strains across different carbon sources in standard MMAT, which contains 25 mM phosphate. On lactate, the *phoT::Tn* mutant had substantially higher membrane potential than WT or the complemented strain (Fig 6C). The *phoT::Tn* mutant also had higher membrane potential on pyruvate or glycerol at pH 5.7 (S8 Fig). At lower phosphate concentrations (up to 200 µM), lactate and phosphate primarily affected cytoplasmic pH. At 25 mM phosphate, however, disrupting PhoT-dependent phosphate transport or signaling was associated with increased membrane potential. Because growth arrest under the conditions in Figs 4, 5, and 6A was associated with lower cytoplasmic pH and largely unchanged membrane potential, these results are consistent with an association between acid growth arrest and reduced PMF.

### The *phoT::Tn* mutant is sensitive to ETC-targeting agents on lactate with high phosphate

Given the effects of lactate and phosphate on PMF, we hypothesized that dysregulation of pH homeostasis and electron transport chain (ETC) functions involved in oxidative phosphorylation contributed to growth arrest. We tested Mtb sensitivity to ETC-targeting and PMF-targeting agents across two carbon sources (pyruvate or lactate), two pH values (7.0 or 6.0), and two phosphate concentrations (0 or 200 µM). We used pH 6.0 rather than pH 5.7 to increase the dynamic range of growth in the 96-well plate format. We tested dose-response curves for thioridazine (TDZ), which targets the non-proton-pumping NDH-2 [26]; clofazimine (CLZ), which targets NDH-2 to generate reactive oxygen species (ROS), among other mechanisms [27–29]; Q203, which targets the cytochrome *bcc::aa*_*3*_ complex [30,31]; bedaquiline (BDQ), which targets ATP synthase [32–34]; nigericin (NIG), a K + /H+ ionophore that dissipates the transmembrane pH gradient [35–37], carbonyl cyanide m-chlorophenyl hydrazone (CCCP), a protonophore that dissipates PMF [38–40]; and valinomycin (VAL), a K+ ionophore that dissipates membrane potential [41–43].

Mtb showed differential sensitivity to several ETC inhibitors depending on growth conditions and the *phoT* mutation, supporting the conclusion that phosphate, lactate, and pH modulated ETC function or PMF (S9 Fig). In lactate with high phosphate, the *phoT::Tn* mutant was more sensitive than WT or the complemented strain to every ETC-targeting agent tested. This sensitivity was not pH-dependent, and Mtb grown on pyruvate was more resistant, indicating that enhanced growth alone did not explain the phenotype. Moreover, the enhanced sensitivity disappeared in lactate with low phosphate, suggesting that lactate and high phosphate together altered ETC function or PMF in the *phoT::Tn* mutant. WT Mtb also showed condition-specific sensitivities consistent with combined effects of lactate and phosphate on ETC function. For example, Mtb was resistant to valinomycin in lactate with 200 µM phosphate at pH 6.0 or 7.0, whereas pyruvate with phosphate or lactate without phosphate promoted valinomycin sensitivity. Clofazimine, nigericin, Q203, and CCCP were more active in lactate at pH 6.0 than at pH 7.0, independent of phosphate, indicating that acidic pH sensitized Mtb to these ETC-targeting drugs. Overall, the *phoT::Tn* mutant was selectively more sensitive to ETC-targeting agents in lactate with high phosphate, consistent with the hypothesis that lactate and phosphate together dysregulate PMF. Alternatively, the differences in sensitivity could be driven by other factors, such as changes in membrane permeability or metabolic constraints.

### Differential regulation of *senX3-regX3,* ESX-5, and ETC genes during acid growth arrest on lactate

Lactate and propionate each arrest growth when provided as a sole carbon source at acidic pH. In a recent study, we found that a *phoPR* null mutant grew on propionate at acidic pH [13]. These observations allowed us to compare transcriptional profiles during acid growth arrest on two nonpermissive carbon sources. We used RNA-sequencing to determine how WT, *phoT::Tn*, and *phoR::Tn* strains responded to acidic pH in the presence of lactate or propionate. We cultured WT Erdman, *phoT::Tn,* and *phoR::Tn* in MMAT buffered to pH 7.0 or pH 5.7 and supplemented with 4 mM lactate or 2 mM propionate. After three days, we extracted RNA and analyzed global transcriptional profiles (S1-S5 Tables).

Comparing WT Mtb cultured on lactate versus propionate at acidic pH revealed shared responses to acidic pH and carbon-source-specific responses. Among genes differentially expressed at pH 5.7 versus pH 7.0, 202 upregulated genes and 205 downregulated genes overlapped between the two carbon sources (S10A Fig). Each carbon source also induced more than 200 unique genes (S10A Fig), demonstrating that growth arrest on propionate differed from growth arrest on lactate. The shared transcriptional signature likely reflected adaptation to acidic pH and included PhoPR-regulated genes (Fig 7A). Genes upregulated only in lactate at acidic pH included genes associated with the electron transport chain, metal uptake, phosphate import, and arginine biosynthesis. In particular, Mtb upregulated numerous cytochromes, including *cydBCD* (Fig 7A).

**Fig 7:**
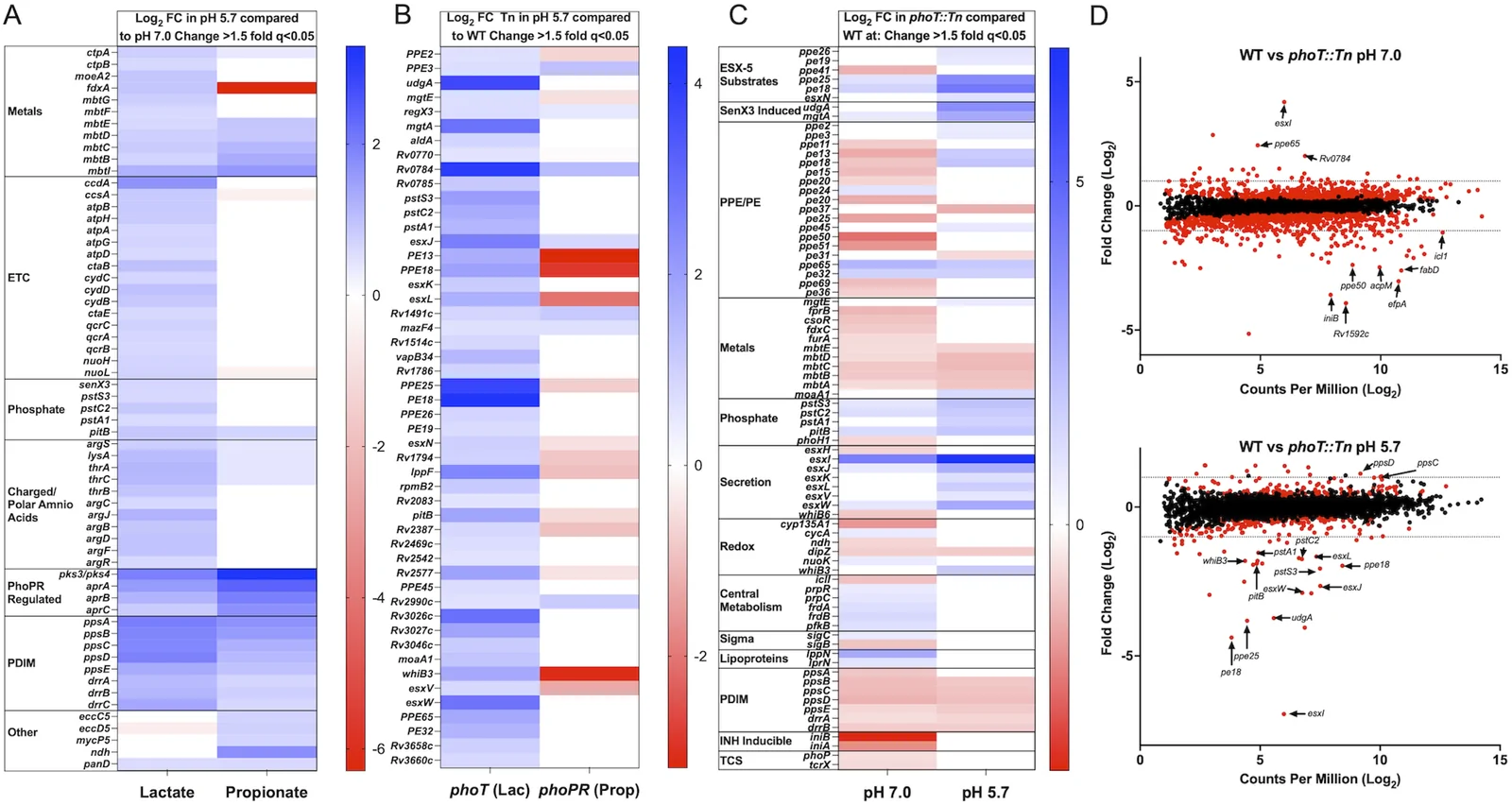
Differential gene expression across carbon sources, pH values, and mutant backgrounds. A. Heat map of genes differentially expressed (fold change > 1.5x and q < 0.05) in WT when exposed to different carbon sources. Genes up and downregulated in lactate at pH 5.7 compared to pH 7.0 on the left. Genes up and downregulated in propionate at pH 5.7 compared to pH 7.0 on the right. B. Heat map of genes differentially expressed (fold change > 1.5x and q < 0.05) in mutant strains capable of growing on their carbon sources at pH 5.7 compared to WT in the same carbon source as each respective mutant at pH 5.7. Genes that change their expression in this comparison indicate how growth affects gene expression compared to growth arrest. C. Heat map of genes differentially regulated (fold-change>1.5, q < 0.05) in the *phoT::Tn* mutant compared to WT at pH 7.0 (left) and pH 5.7 (right). Upregulation in the *phoT::Tn* mutant is indicated by blue and downregulation in the mutant is indicated by red. D. Magnitude/amplitude plots of average Log_2_ counts per million (CPM) and Log_2_ fold-change of the *phoT::Tn* mutant grown at pH 7.0 and pH 5.7 compared to the WT grown at pH 7.0 and pH 5.7.

Next, we compared genes associated with growth versus growth arrest on each carbon source by comparing *phoT::Tn* with WT at pH 5.7 in lactate and *phoR::Tn* with WT at pH 5.7 in propionate. This analysis identified carbon-source-specific transcriptional changes associated with growth. The upregulated gene sets showed little overlap (S10B Fig), indicating that suppression of growth arrest on lactate and propionate involved different processes. Genes upregulated only in the *phoT::Tn* mutant included several SenX3-RegX3-regulated genes, such as *mgtA, udgA, pstA1, pstC2*, and *pe19* (Fig 7B). Notably, electron transport chain genes were unchanged. The persistence of the lactate-associated ETC transcriptional signature in *phoT::Tn* mutant suggests that suppression of growth arrest does not require its reversal.

We next identified genes differentially regulated in *phoT::Tn* relative to WT at pH 5.7 or pH 7.0. The two comparisons showed little overlap (S10C Fig), suggesting that *phoT* had limited pH-independent regulatory effects. Genes upregulated in *phoT::Tn* at both pH values included phosphate-related genes, SenX3-RegX3-regulated genes, and ESX-5 substrate genes (Fig 7C-7D). These findings indicated that SenX3-RegX3 was activated in the *phoT::Tn* mutant and increased expression of phosphate transport machinery. Comparing pH 5.7 with pH 7.0 in WT and *phoT::Tn* also showed induction of the PhoPR regulon at acidic pH in both strains (S11A-S11D Fig). Both strains upregulated phosphate-related genes. Overall, the transcriptional signatures indicate that acid growth arrest on lactate modulates SenX3-RegX3, ESX-5, and the ETC.

### Growth rescue on lactate depends on the SenX3-RegX3 two-component regulatory system

SenX3-RegX3 is a two-component regulatory system in Mtb in which SenX3 is the membrane-bound histidine kinase and RegX3 is the response regulator [44–46]. High phosphate inhibits SenX3 autophosphorylation; under low-phosphate conditions, SenX3 autophosphorylates and phosphorylates RegX3, which regulates genes involved in phosphate starvation and other pathways [44,46,47]. Our transcriptional profiling data suggested that SenX3-RegX3 contributed to growth on lactate. Using a series of null mutant strains that were generated previously [16,48–50], along with a newly generated ∆*phoT* mutant and complemented strain (S12 Fig), we tested whether PhoT, PstA1, PE19, and RegX3 mediated the phosphate-, lactate-, and pH-dependent phenotypes. We used the independent ∆*phoT* mutant to confirm that loss of PhoT caused the observed phenotype without relying solely on the original transposon mutant. In acidified medium containing lactate as the sole carbon source, WT and complemented strains grew without phosphate but not with phosphate (Fig 8A). In contrast, the *∆phoT* mutant grew to an OD_600_ of ~0.4 in the presence of phosphate (Fig 8A). The *∆phoT* mutant did not grow when phosphate was absent, possibly because it lacked stored phosphate that WT accumulated during initial growth in rich medium. Because PhoT is predicted to function with the PstSCA transport system during phosphate translocation, we hypothesized that disrupting another component of this system would phenocopy ∆*phoT*. We therefore tested a ∆*pstA1* mutant [16]. PstA1 is a transmembrane component of the phosphate transport system. The ∆*pstA1* mutant phenocopied ∆*phoT*: it grew to an OD_600_ of ~0.3 with phosphate but did not grow without phosphate (Fig 8B). These results show that the phosphate-starvation and enhanced-growth-on-lactate phenotype depends on the PhoT-PstSCA complex, consistent with studies of glycerol in modified 7H9 medium [17].

**Fig 8:**
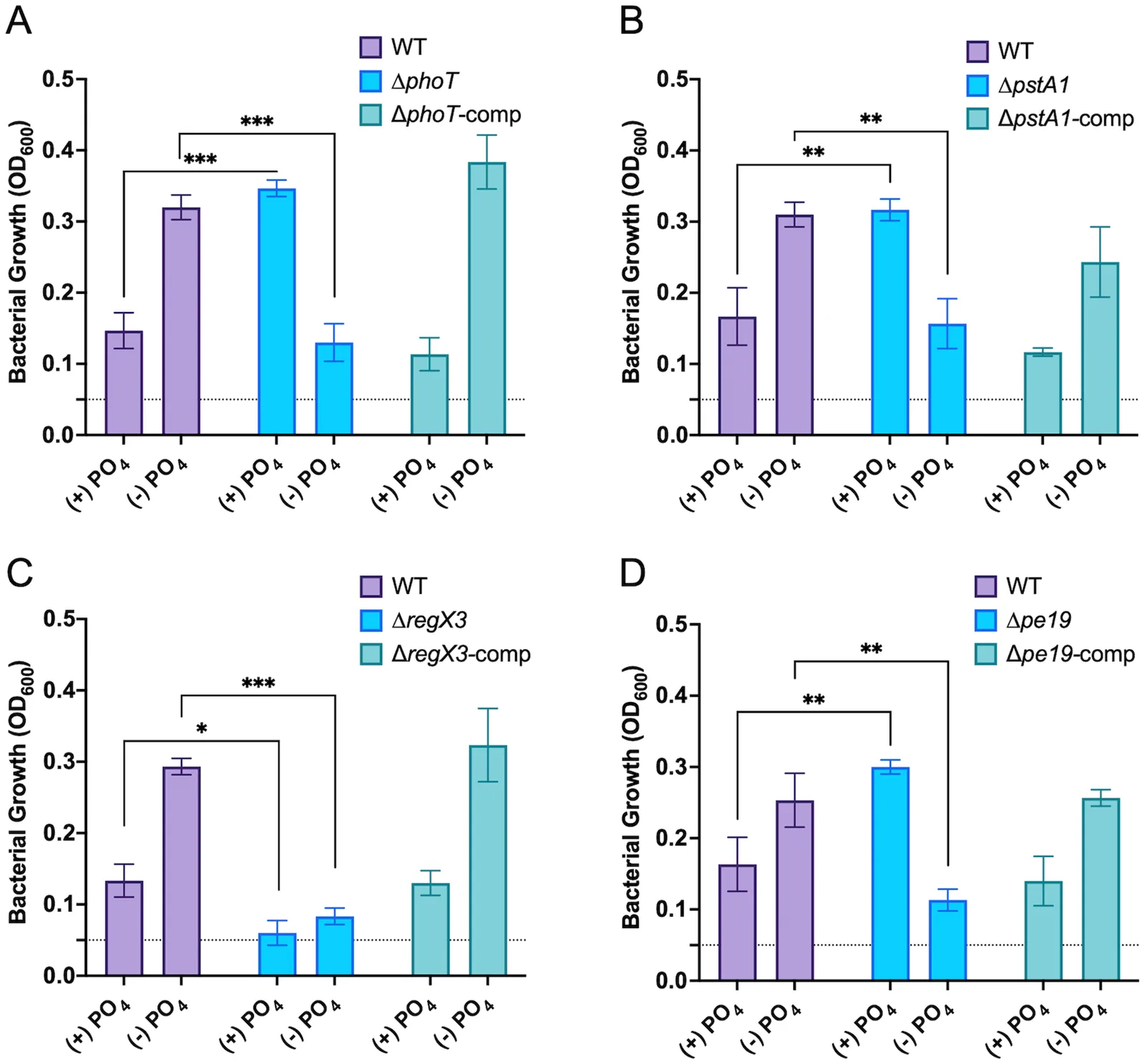
Components of the phosphate-response pathway differentially regulate growth on lactate. A. WT (purple), ∆*phoT* (light blue), and ∆*phoT* complemented strain (teal) grown in MMAT pH 5.7 either lacking phosphate completely or with the high 25 mM concentrations of phosphate in MMAT, 4 mM lactate was used. Statistical significance was determined using an unpaired t-test (***, p < 0.001). B. WT (purple), ∆*pstA1* (light blue), and ∆*pstA1* complemented strain (teal) grown in similar medium described above. Statistical significance was determined using an unpaired t-test (**, p < 0.005). C. WT (purple), ∆*regX3* (light blue), and ∆*regX3* complemented strain (teal) grown in similar medium as described above. Statistical significance was determined using an unpaired t-test (*, p < 0.05 ***, p < 0.001). D. WT (purple), ∆*pe19* (light blue), and ∆*pe19* complemented strain (teal) in similar medium as described above. Statistical significance was determined using an unpaired t-test (**, p < 0.005). All four experiments were performed at least twice with similar results. The error bars represent standard deviations of three technical replicates.

We hypothesized that the phosphate-starvation-sensing two-component system SenX3-RegX3 mediated phosphate-starvation-dependent growth on lactate. We therefore compared WT, ∆*regX3*, and the complemented strain under the conditions used for ∆*phoT* and ∆*pstA1*. The ∆*regX3* strain did not grow on lactate with or without phosphate (Fig 8C), demonstrating that growth on lactate under phosphate-limited conditions requires SenX3-RegX3. PPE and PE proteins have recently been implicated in nutrient uptake [12,51–54], and PE19 specifically contributes to phosphate-dependent growth arrest on glycerol [17]. We therefore hypothesized that *pe19*, a member of the SenX3-RegX3 regulon [50], contributed to growth on lactate. The ∆*pe19* mutant phenocopied ∆*phoT* and ∆*pstA1* where it grew to an OD_600_ of ~0.3 with phosphate but not without phosphate (Fig 8D). These data suggest that PE19 may regulate phosphate sensing, permeability, or uptake pathways that promote growth arrest on lactate at acidic pH.

### Phosphate limitation promotes growth on glycerol, glucose, and lactate

A recent study showed that a ∆*phoT* strain grew on glycerol at pH values as low as 5.0 *[17]*, whereas our *phoT::Tn* mutant did not grow on glycerol at pH 5.7. The studies differed in Mtb strain (Erdman versus H37Rv) and medium (MMAT versus carbon-depleted 7H9). Because MMAT is less complex than 7H9, we repeated the assay using the medium described by Healy *et al*. [17] with our strains and growth conditions. In modified 7H9, ∆*phoT* and *phoT::Tn* grew robustly on glycerol but did not grow on glucose (S13A-S13B Fig), replicating the findings of Healy *et al*. These data indicate that a component present in carbon-depleted 7H9 but absent from MMAT permits *phoT* mutant strains to grow on glycerol. We next asked whether depleting phosphate from MMAT altered WT growth on different nonpermissive carbon sources. Under low-phosphate conditions, WT grew more on glucose, glycerol, and lactate (S13C Fig). The different carbon-source growth profiles of the *phoT::Tn* mutant (Fig 2A) and phosphate-depleted WT (S13C Fig) show that *phoT::Tn* did not fully phenocopy a low-phosphate environment and suggest that more complex interactions among pH, phosphate, and carbon source controlled growth.

## Discussion

Mtb establishes a state of nonreplicating persistence on lactate at acidic pH, and genetic mutation overcomes this growth arrest. This finding supports our “acid fasting” hypothesis that metabolic restrictions cause growth arrest at acidic pH [9]. Dominant mutations in *ppe51* overcome growth arrest on glycerol by increasing glycerol uptake [12], loss-of-function mutations in *phoPR* overcome growth arrest on propionate by rerouting carbon from lipid synthesis to central metabolism [13], and, as shown here, limiting phosphate overcomes growth arrest on lactate. These three distinct mechanisms suggest that Mtb has evolved to constrain growth in acidic environments and that specific carbon sources and other nutrients, such as phosphate, regulate persistence versus growth.

Mtb encounters acidic pH after macrophage phagocytosis and possibly in granulomatous tissues, where measured pH values range from 5.0 to 7.2 [55]. In the guinea pig infection model, lactate accumulates in granulomatous tissue as infection progresses [56]. Stanley *et al*. showed that ongoing accumulation of mutations and evolution of the lactate dehydrogenase gene *lldD2* in Mtb confers a selective advantage during adaptation to host-associated stresses [57]. Mtb infection also induces the Warburg effect in mouse lungs, causing inflamed tissue surrounding infection sites to undergo aerobic glycolysis and produce lactate [58–60]. In a rabbit model of *Staphylococcus aureus* lung infection, tissue lactate concentrations can reach 15 mM [61]. L-lactate is the predominant host isomer and was the isomer used in this study. A recent study suggested that Rv1257c functions as a D-lactate dehydrogenase and may allow Mtb to use D-lactate in the host; the same study found that a *ppe3* transposon mutant grew poorly on both L- and D-lactate *in vitro* [62]. Lactate also acts as an immunomodulatory signal through autocrine and paracrine mechanisms [63–65]. Because measuring phagosomal metabolites during active infection remains difficult, the availability of lactate and phosphate to intracellular Mtb is unknown. Nevertheless, abundant lactate in infected tissue and potentially limited phosphate in the phagosome suggest that Mtb may encounter this combination of signals during infection, particularly as an extracellular bacterium in necrotic tissue. Integrating lactate concentration, phosphate availability, and pH may allow Mtb to sense nutrient availability and the immune environment and regulate growth.

The effects of lactate on bacterial physiology are well documented because of its importance in industrial processes [66–68]. We hypothesized that lactate contributed to growth arrest through stresses similar to those produced by excessive lactate accumulation in lactic acid bacteria. These stresses include cytoplasmic acidification [21], membrane permeabilization and reduced membrane potential [22], and the resulting decrease in PMF. Mtb tightly regulates its cytosolic pH [5,8,69], and deviations from the typical range of ~7.2-7.4 indicate stress. The ETC oxidizes reduced cofactors and pumps protons to generate the gradient that drives ATP synthesis, linking ETC function closely to PMF. We hypothesize that the cytoplasmic acidification observed here may have contributed to growth arrest without killing Mtb because lethal cytoplasmic pH values in *Mycobacterium smegmatis* and *Mycobacterium bovis* BCG range from 5.5 to 6.0 [24], well below the values that we measured. Our data associate acidic pH, lactate, and phosphate with cytoplasmic acidification and PMF stress, which may contribute to growth arrest at acidic pH.

We propose a two-part model involving physiological stress and nutrient uptake to explain Mtb growth arrest on lactate at acidic pH. In the first part, the cytoplasmic pH stress model, lactate and phosphate impose physiological constraints as Mtb maintains pH homeostasis, PMF for oxidative phosphorylation, and protein folding. When protonated lactic acid enters the cell, dissociation can release a proton into the cytoplasm; phosphate uptake may also alter cytoplasmic pH or the energetic demands of pH homeostasis (Figs 4 and 5). When lactate is the sole carbon source, Mtb may export protons to maintain pH homeostasis, which would increase membrane potential. Inactivating *phoT* may reduce phosphate import or alter phosphate-responsive signaling, thereby relieving cytoplasmic acidification and allowing the bacterium to maintain a higher membrane potential. A more neutral cytoplasmic pH and higher membrane potential may preserve sufficient PMF for oxidative phosphorylation and growth. We speculate that, above specific lactate and phosphate thresholds, cytoplasmic pH may fall below ~6.8 and PMF may become insufficient to support oxidative phosphorylation. Our RNA-sequencing data were consistent with this model: ETC-associated genes were upregulated in lactate at acidic pH but not in propionate. At moderately acidified cytoplasmic pH values, such as 6.6, the activity or stability of pH-sensitive metabolic enzymes may also be impaired [20,70–75]. The RNA-sequencing data also showed upregulation of the arginine biosynthesis pathway. In *E. coli*, arginine serves as a proton sink during cytoplasmic acid stress [76], and arginine decarboxylation consumes a proton and produces agmatine for export [76]. Together, these data suggest that exposure to acidic pH, lactate, and phosphate can acidify the cytoplasm and constrain growth. Changing the relative abundance of lactate or phosphate, or changing extracellular pH, can mitigate this acidification.

In the second part of the model, restricted phosphate uptake through *phoT* mutation or phosphate limitation activates SenX3-RegX3, which upregulates ESX-5 genes and promotes export of PPE/PE proteins that may remodel the cell envelope or facilitate nutrient uptake (Fig 9). Thus, either mutation of the PstSCA*-*PhoT phosphate transport system or reduced extracellular phosphate may lower intracellular phosphate and activate SenX3-RegX3 [44,46,47]. Tischler *et al*. showed that deletion of *pstA1,* which encodes a transmembrane component of PstSCAB, constitutively activates SenX3-RegX3 signaling [16]. Because the orphan ATPase PhoT putatively interacts with PstS3, PstC2, and PstA1, we propose that disrupting *phoT* similarly activates SenX3-RegX3. The SenX3-RegX3 regulon controls phosphate-associated genes [16,44,77] and activates the redox sensor WhiB3 [78] which our transcriptional profiling showed was upregulated (Figs 7B-7C, S11A). Deleting *pstA1* constitutively activates SenX3-RegX3 and induces *udgA*, *mgtA*, and other genes [16,48,50]; we observed the same genes upregulated in the *phoT::Tn* mutant at pH 5.7 and pH 7.0 (Fig 7B). Our data therefore showed that a ∆*phoT* mutant phenocopied the ∆*pstA1* strain. Because the ∆*regX3* mutant did not grow on lactate with or without phosphate, RegX3 was required to overcome growth arrest on lactate. One or more genes downstream of SenX3-RegX3 may permit growth on lactate by alleviating lactate-associated pH stress or maintaining cytoplasmic pH and PMF. Alternatively, a PE/PPE protein in the regulon may promote uptake of a nutrient, such as magnesium or another metal, that alleviates pH stress. The ∆*pe19* mutant also phenocopied ∆*phoT* and ∆*pstA1* (Fig 8D), suggesting that PE19 contributes to transport of phosphate or another nutrient required for growth. Overall, disrupting *phoT* or *pstA1* was associated with SenX3-RegX3 activation, and a RegX3-regulated protein may permit growth on lactate at acidic pH.

**Fig 9:**
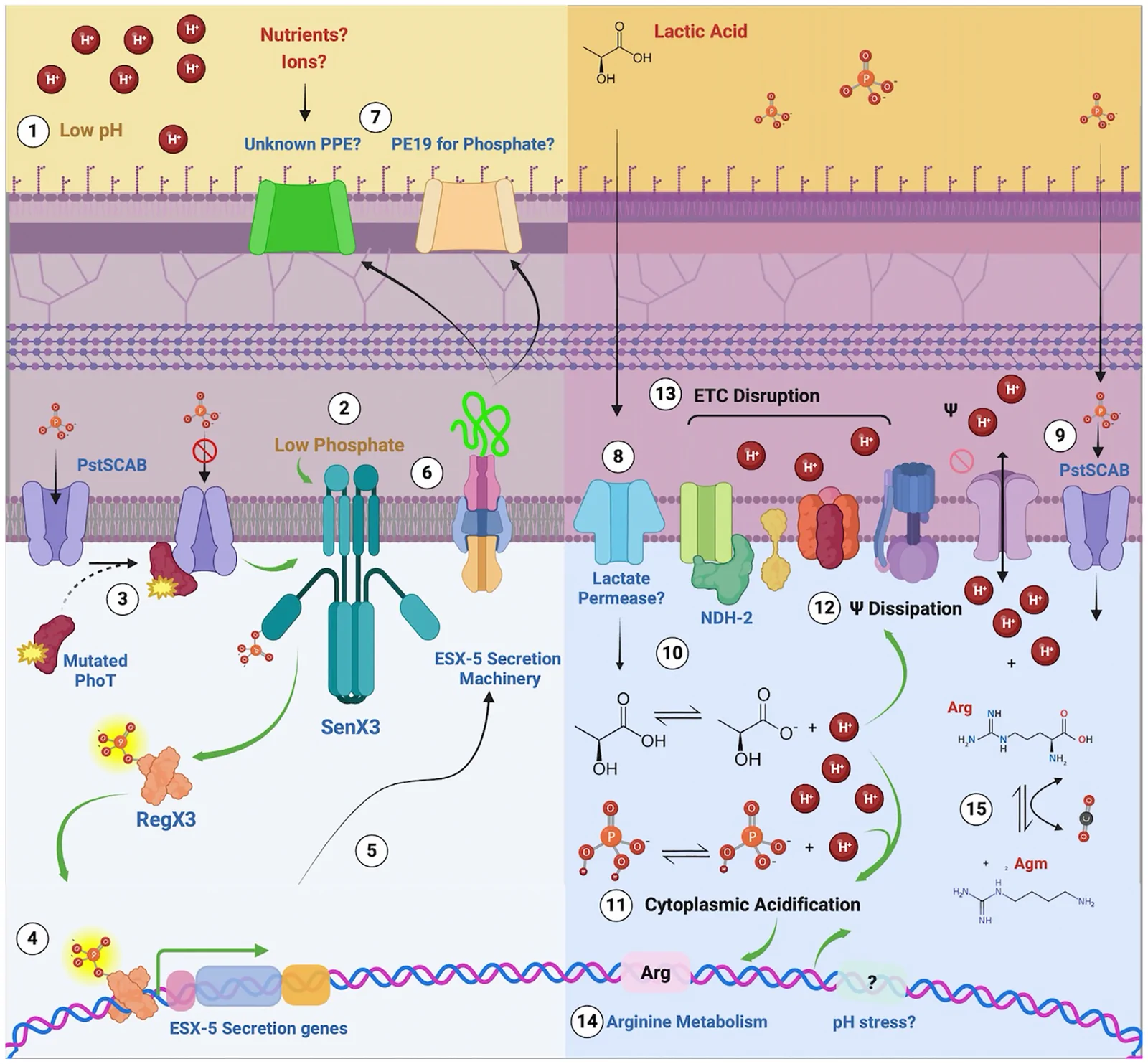
Proposed model for the impact of lactate and phosphate on Mtb growth at acidic pH. At acidic pH (~5.7) (1) Mtb tightly regulates cytoplasmic pH to around 7.2-7.4. Under low phosphate conditions (2) or following disruption of the PstSCA-PhoT system (3) SenX3-RegX3 signaling is activated, resulting in the phosphorylation of the response regulator RegX3 (4), and the expression of genes in its regulon, including the components of the ESX-5 secretion system. Some unknown genes in the RegX3 regulon might also be involved in alleviating the pH stress induced by acidic growth conditions or an agent acidifying the cytoplasm. The ESX-5 system is assembled and inserted into the cytoplasmic membrane (5), where it exports several proteins to the mycomembrane (6), some of which can be PPE and PE proteins (e.g., PE19), which may play a role in membrane permeability, nutrient acquisition, transport regulation, or ion balance (7). A putative permease can then transport lactate from the pseudoperiplasmic space into the cytoplasm (8). Alternatively, protonated lactic acid may diffuse passively across the membrane in acidic environments. Lactic acid dissociation can release protons into the cytoplasm (10), and phosphate uptake may further affect pH homeostasis or its energetic demands (9), together promoting cytoplasmic acidification (11). Additionally, maintenance of cytoplasmic pH homeostasis on lactate may result in proton export modestly increasing membrane potential (𝚿) (12), which may also be altered by disruption of PhoT-dependent phosphate transport or signaling. Because cytoplasmic pH contributes to the proton gradient used for oxidative phosphorylation, cytoplasmic acidification may reduce PMF and impose ETC stress (13). As a countermeasure to cytoplasmic acidification, Mtb upregulates genes responsible for the synthesis of arginine (14), an amino acid that can act as a sink for excess protons (15). Green arrows indicate positive influence (i.e., increase signaling or expression). Created in BioRender. Abdalla, **B.** (2026) https://BioRender.com/onfcs7u.

We previously performed RNA-seq analysis under similar conditions using WT CDC1551 exposed to pyruvate or glycerol at acidic or neutral pH [8], which allowed us to identify lactate-specific adaptations to acidic pH. A limitation of this comparison is that the lactate data came from Erdman, whereas the glycerol and pyruvate data came from CDC1551. Among 529 genes upregulated in lactate at acidic versus neutral pH, 292 were specific to lactate (S14 Fig). Cytochrome biogenesis genes *ccdA*, *ccsA*, *ctaE*, and *qcrCAB* and phosphate transport genes *pstS3*, *pstC2*, and *pstA1* were upregulated only in lactate at acidic versus neutral pH, not in glycerol or pyruvate at acidic pH. This pattern further suggests that lactate causes PMF stress requiring cytochrome biogenesis and links phosphate transport and signaling to lactate metabolism. Consistent with this model, cytochrome bd expression in *Bacillus subtilis* is linked to lactate dehydrogenase expression during microaerobic growth [79]. Notably, Region of Difference 1 (RD1) genes, which contribute to pathogenesis [80], were downregulated only in lactate at acidic pH, not in pyruvate or glycerol. These data suggest that carbon source availability differentially regulates virulence factors. Together, these findings support a model in which Mtb integrates acidic pH and carbon source availability to regulate ETC, phosphate transport, metal homeostasis, and virulence genes.

Concurrent with our study, Healy *et al.* showed that ∆*phoT* and ∆*pstA1* mutants grew on glycerol at acidic pH [17]. Although our *phoT::Tn* mutant did not show this phenotype in MMAT (Fig 2A), it grew on glycerol in modified 7H9, replicating their result [17]. MMAT and 7H9 differ substantially, and future work should identify which medium components determine lactate specificity in MMAT and growth of the *phoT::Tn* mutant. Depleting phosphate from MMAT also allowed WT Erdman to grow on glycerol at pH 5.7. This result suggests that the inability of *phoT::Tn* to grow on glycerol in MMAT may reflect a hypomorphic phenotype in which residual or compensatory phosphate uptake through other systems persists. Defining these interactions should clarify how Mtb fine-tunes growth on specific carbon sources at acidic pH.

## Materials and methods

### Bacterial strains and media

Experiments were performed using Mtb Erdman grown at 37°C in standing, filter-vented T25 or T75 tissue culture flasks unless otherwise specified. Cultures were maintained in 7H9 medium supplemented with 10% OADC, 0.5% glycerol, and 0.05% Tween-80. Experiments performed in minimal defined MMAT medium as described by Lee *et al*, 2013: 1 g L^−1^ KH_2_PO_4_, 2.5 g L^−1^ Na_2_HPO_4_, 0.5 g L^−1^ (NH_4_)_2_SO_4_, 0.15 g L^−1^ asparagine, 10 mg L^−1^ MgSO_4_, 50 mg L^−1^ ferric ammonium citrate, 0.1 mg L^−1^ ZnSO_4_, 0.5 mg L^−1^ CaCl_2_ and 0.05% Tyloxapol. The media was buffered to pH 5.7 or 7.0 using 100 mM MES or MOPS respectively [81]. In the media lacking phosphate, KH_2_PO_4_ and Na_2_HPO_4_ were omitted, then supplemented back in the form of KH_2_PO_4_. To replicate the medium described by Healy et al. [17], we prepared 7H9 containing 5 g L^-1^fatty-acid-free albumin, 3.0 mg L^-1^ of catalase, buffered to pH 5.0 with MES. For the two media, 0.2% glycerol or 0.2% dextrose was added, respectively. In all experiments, cultures were grown to mid-log phase, then washed and seeded in experimental growth medium (MMAT high phosphate, MMAT low phosphate, or modified 7H9) at an OD_600_ of 0.05.

Growth experiments performed with varying phosphate sources, bacteria were grown to mid-log, washed and resuspended in modified MMAT. All growth conditions were buffered to pH 5.7 and contained 4 mM lactate. The conditions were full MMAT media, MMAT media with no phosphate sources, MMAT with only 1 g l^−1^ KH_2_PO_4_, and MMAT with only 2.5 g l^−1^ Na_2_HPO_4._

The Mtb Erdman ∆*pstA1*, ∆*pstA1*-comp, ∆*regX3*, ∆*regX3*-comp, ∆*pe19* and ∆*pe19*-comp strains were previously described [16,50]. The Mtb Erdman ∆*phoT* mutant was made by allelic exchange with pJG1100-∆*phoT*. Regions of the *Mtb* Erdman genome ~850 bp 5’ and 3’ of *phoT* were amplified by PCR and cloned in pJG1100 between the PacI and AscI sites and Sanger sequenced. The pJG1100-∆*phoT* plasmid was electroporated in *Mtb* Erdman and transformants were selected on 7H10 agar with kanamycin (15 µg/ml) and hygromycin (50 µg/ml). Transformants were then grown without antibiotics and plated on 7H10 with 2% sucrose to counterselect the pJG1100 plasmid as described [16]. The ∆*phoT* mutant was confirmed by PCR and Southern blotting. Production of the virulence lipid phthiocerol dimycocerosate (PDIM) by the ∆*phoT* mutant was verified by thin layer chromatography of ^14^C propionate-labeled cell wall lipids as described [16]. Primer sequences are available upon request.

The complementation plasmids for the *phoT::Tn* strain were generated using a single-copy chromosomal insertion vector (pMV306) and an episomal overexpression vector (pVV16). The WT gene *phoT* was cloned into pMV306 using Gibson cloning, taking approximately ~1000 bp upstream to include the native promoter. The complete pMV306::*phoT* construct was electroporated into *phoT::Tn* strains and selected for on 7H10 plates with zeocin (25 µg/mL). WT *phoT* was cloned into pVV16 using Gibson cloning. For the pVV16 construct, the *phoT* gene was cloned under the *hsp60* promoter. The complete pVV16::*phoT* construct was electroporated into *phoT::Tn* strains and selected on 7H10 agar with hygromycin (50 µg/mL).

### Construction of transposon library

A Mtb Erdman transposon library was generated using the φMycoMar T7 phage as previously described [82,83]. Wild type Mtb was grown to mid-log phase in 50 mL of 7H9 supplemented with 10% OADC and 0.05% Tween-80, after which bacteria were washed and incubated in 4.5 mL of MP buffer (50 mM Tris-HCl pH 7.5, 150 mM NaCl, 10 mM MgSO_4_, and 2 mM CaCl_2_) with 1x10^11^ PFU φMycoMar T7 at 37°C for 4 hours. The Mtb-phage mix was then split between 8 or more 15 cm 7H10 plates containing 25 µg/mL kanamycin and incubated for three to six weeks at 37°C. Libraries totaling ~100,000 mutants were collected in 2–4 separate pools and stored at -80°C for subsequent use.

### Genetic selections

To select for spontaneous mutants, Mtb Erdman was grown to an OD_600_ of 0.6-1.0, spun down, and resuspended in MMAT media buffered to pH 5.7. Mtb cells were calculated for 10^9^ CFU and plated across 4 MMAT agar plates buffered to pH 5.7 supplemented with 4 mM lactate. Plates were incubated at 37°C for over 12 weeks without any isolated colonies appearing.

To select for transposon mutants, an Mtb Erdman transposon library consisting of >100,000 mutants was thawed and grown for 24 hours in 10 mL unbuffered 7H9 supplemented with 10% OADC and 0.05% Tween-80 to allow for initial growth. The OD_600_ was measured on a spectrophotometer, calculated for 10^9^ CFU, and plated on 4 MMAT agar plates buffered at pH 5.7 and supplemented with 4 mM lactate. Plates were incubated for 4–8 weeks at 37°C after which 20 isolated colonies were randomly chosen for follow-up growth studies. Isolated colonies were confirmed for growth at acidic pH in liquid culture (MMAT + 4 mM lactate buffered to pH 5.7). The inverse PCR technique [18] was used initially on two colonies to identify transposon insertion sites but was later replaced using whole genome sequencing (WGS) combined with the BLAST-like alignment tool (BLAST) [84] for more robust results. For WGS, genomic DNA was extracted and sequenced using the Illumina MiSeq and a 200 Mbp (1.33M reads) sequencing package. Following the run, reads were trimmed and the variant caller breseq [85] was used to align and compare sequencing data against the Erdman or CDC1551 reference genomes. BLAST was used to map the φMycoMar T7 transposon sequence onto assembled reads and find the transposon insertion breakpoints. The NCBI reference number for the mapping of the Erdman Tn insertion sites is NZ_KK339487.1.

### Lactate and phosphate dose response checkerboard assay

The checkerboard experiment was performed in black-sided, clear bottom 96-well plates. Medium was seeded with Mtb at an OD_600_ of 0.05 in wells with a 2.5-fold dilution series KH_2_PO_4_ (0 µM – 200 µM) and a 2.5-fold dilution series of lactate (0 µM – 40 mM) examining all combinations. The plates were then incubated for 21 days. Before taking readings in the plate reader, the cells were resuspended to ensure homogeneity. ODs were normalized to percent-of-maximum using wild-type minimum and maximum growth values. Each condition was conducted in triplicate.

### Cytoplasmic pH assay

Cytoplasmic pH was measured using the protocol described by Purdy *et al.*, using the pH-sensitive dye CMFDA [23]. Mtb was grown in rich 7H9 to mid-log phase, cells were pelleted and resuspended in fresh 7H9 in 500 µL at an OD600 of 0.5. 4 µL of 1 mg mL^-1^ CMFDA dye was added to each sample and incubated for 2 hours at 37°C. Samples were then washed twice in 7H9 to remove excess CMFDA. After the last wash, the cells are resuspended in MMAT with phosphate omitted, then transferred to a 96-well plate where lactate and phosphate were added, fluorescence was then measured with excitations of 450 nm and 490 nm and emission at 520 nm for the course of 60 minutes. A standard curve was generated using Mtb in buffered medium treated with 10 µM nigericin.

### Measuring membrane potential

Mtb was grown in rich 7H9 to mid-log phase, cells were pelleted and resuspended in fresh 7H9 in 500 µL at an OD600 of 0.5. The fluorescent dye DiOC_2_ was added to a final concentration of 30 µM. Cells were labeled for 30 minutes at room temperature. The cells were then washed twice in 7H9 to remove excess DiOC_2_. After the final wash, cells were resuspended in MMAT with different modifications. For Fig 6A-6B, modified MMAT medium, pH 5.7, with either no potassium phosphate or 200 µM potassium phosphate was used with increasing amounts of lactate (1, 4, and 10 mM). For Figs 6C and S8D, standard MMAT medium, pH 5.7, containing both sodium and potassium phosphate were used with varying carbon sources (4 mM lactate, 10 mM glycerol, or 10 mM pyruvate). Lastly, S7 Fig, modified MMAT, pH 5.7, 4 mM lactate, and varying phosphate sources; either both sodium phosphate and potassium phosphate, only sodium phosphate, only potassium phosphate, or no phosphate, as described in the growth experiments above. Cells were then transferred to a 96-well plate. Carbonyl cyanide m-chlorophenyl hydrazone (CCCP) was used as a depolarizing agent at a final concentration of 50 µM. Emission spectra were read at 610 and 515 nm after exciting at 485 nm over the course of 60 minutes.

### ETC inhibitor dose response assays

Mtb was grown up to mid-log phase, cells were washed and then seeded in a black-sided, clear-bottom 96 well plate at an OD of 0.05. Plates were incubated at 37°C in a plastic bag containing a wet paper towel to maintain humidity and prevent evaporation. Plates were read after 10 days of incubation. For each strain a rifampicin and DMSO treatment were included as positive and negative controls that were later used for normalization.

### Transcriptional profiling and data analysis

Initial Mtb cultures (wild type, *phoT*::*Tn* mutant 2.4, and *phoR::Tn*) were grown in 7H9 buffered media at 37°C and 5% CO_2_ in T-25 vented flasks. Cultures were expanded out to 75 mL 7H9 buffered media. Once mid-log growth phase was reached a second time, cultures were spun down and pre-adapted for three days in MMAT (pH 7.0 + glycerol) to account for metabolite carryover. Experimental cultures were inoculated at a starting OD_600_ of 0.6 in 8 mL of MMAT buffered media including 4 mM lactate or 2 mM propionate. Treatment conditions examined include i) wild type Mtb at pH 5.7 with lactate, ii) wild type Mtb at pH 5.7 with propionate, iii) wild type Mtb at pH 7.0 with lactate, iv) wild type pH 7.0 with propionate, v) *phoT::Tn* at pH 5.7 with lactate, and vi) *phoT::Tn* at pH 7.0 with lactate, vii) *phoR::Tn* at pH 5.7 with propionate, viii) *phoR::Tn* at pH 7.0 with propionate. Each treatment condition was performed with two biological replicates and incubated for three days after which total bacterial RNA was extracted as previously described using the TRIzol method and then an Qiagen RNeasy kit (ID: 74104) [8,86]. RNA quantity and quality was assessed by Qubit Fluorometer using the Qubit RNA BR kit (ID: Q10210) and Agilent 2100 Bioanalyzer, respectively. DNA was depleted using the Invitrogen TURBO DNA-free kit (ID: AM1907). Finally, the RNA library was sequenced using a NovaSeq 6000. Raw sequencing data was analyzed using SPARTA (ver. 1.0) [87]. Genes were filtered based on > 1.5-fold differential gene expression and q < 0.05. Transcriptional profiles are deposited in the GEO Database (accession number GSE301034).

## Supporting information

S1 Fig Growth arrest on lactate can be overcome through pyruvate supplementation or genetic mutation. (PDF)

S2 Fig Growth arrest in lactate can be overcome in both WT H37Rv and WT Erdman by depleting phosphate. (PDF)

S3 Fig Mtb arrests growth regardless of counterion used for supplementation with phosphate. (PDF)

S4 Fig Growth arrest on lactate is pH-dependent. (PDF)

S5 Fig Cytoplasmic pH is not impacted in strains exposed to lactate and phosphate at neutral pH. (PDF)

S6 Fig Lactate and phosphate at low concentrations have modest impacts on membrane potential. (PDF)

S7 Fig Potassium phosphate has a negative impact on membrane potential in WT Mtb. (PDF)

S8 Fig The *phoT::Tn* mutant has higher membrane potential in pyruvate and glycerol. (PDF)

S9 Fig Dose-response curves of Electron transport chain-targeting agents across different growth conditions and genetic backgrounds. (PDF)

S10 Fig Venn diagrams depicting significant overlaps between comparisons of differential gene expression. (PDF)

S11 Fig Genes differentially regulated when comparing pH 5.7 to 7.0 in the *phoT::*Tn mutant or WT Mtb. (PDF)

S12 Fig *∆phoT* mutant validation. (PDF)

S13 Fig *phoT* mutants grow on glycerol at acidic pH on modified 7H9 medium. (PDF)

S14 Fig Subsets of downregulated and upregulated genes are specific to lactate at acidic pH. (PDF)

S1 Raw gel Uncropped images. (PDF)

S1 Table Gene expression tables of Mtb grown in lactate at pH 5.7 as compared to pH 7.0. (XLSX)

S2 Table Gene expression tables of *phoT*::Tn grown in lactate at pH 5.7 compared to WT grown in lactate at pH 5.7. (XLSX)

S3 Table Gene expression tables of *phoT*::Tn grown in lactate at pH 5.7 compared 7.0. (XLSX)

S4 Table Gene expression tables of *phoR*::Tn grown in propionate at pH 5.7 compared to WT grown in propionate at pH 5.7. (XLSX)

S5 Table Gene expression tables of WT grown in propionate at pH 5.7 compared to pH 7.0. (XLSX)

S1 Text Supplemental figure legends. (PDF)
