# Supplementary material for: Combined lactate- and phosphate-dependent cytoplasmic acidification is associated with Mycobacterium tuberculosis growth arrest at acidic pH
Source: PLoS Genet. 2026 Oct 9;22(10):e1012331. doi: 10.1371/journal.pgen.1012331 (PMC13716317; doi:10.1371/journal.pgen.1012331)
Supplement: Supplementary file 10 [file pgen.1012331.s010.pdf]

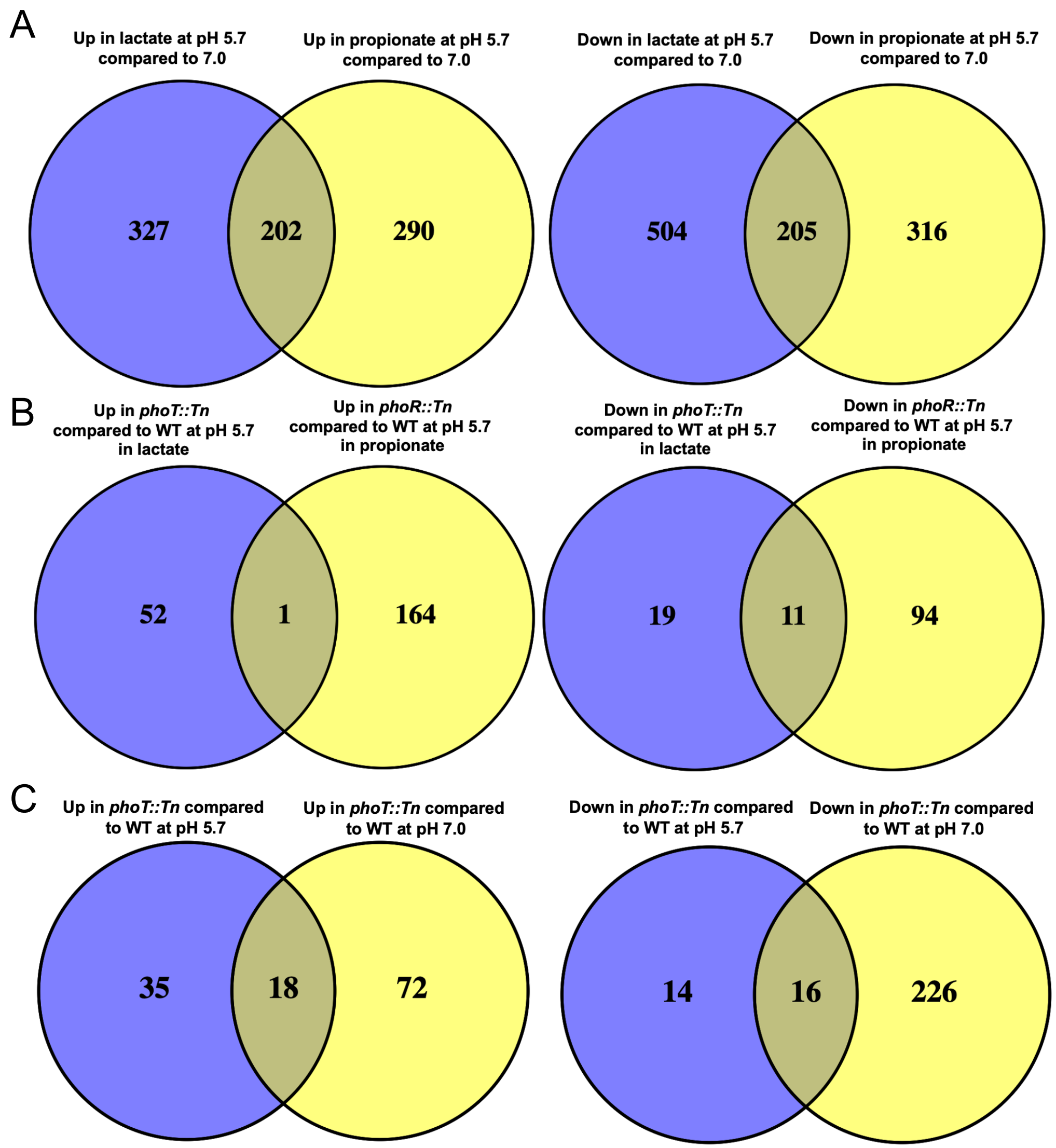

Supplemental Figure 10
